# Cancerous Disease of the Lungs

**Published:** 1878-09

**Authors:** J. Fowler


BUFFALO

### Medical and Surgical Journal.

VOL. XVIII. SEPTEMBER, 1878. NO. 2.

### Original Communications.

———:o:———

*ART. I.—Cancerous Disease of the Lungs.* Report of a case before Buffalo Medical Association. By J. FOWLER, M. D.

Mrs. B. was nearly 64 years old, and a widow for five years. She was the mother of four children, three of whom died of pulmonary tuberculosis when aged respectively, 18, 21 and 30 years.

She stated that she had always enjoyed good health until my first visit, April 11; except at irregular intervals during the last three or four years had experienced some pain in left side extending to her hip. It caused her little or no inconvenience, nor did she think it worth while to seek medical advice. Her father died at the age of seventy-four or five of pulmonary disease, and her mother of pulmonary phthisis, aged eighty-four years.

At my first visit, April 11th, I found her suffering from pain in the left infra-axillary region, with a considerably febrile disturbance and chills, said she, "my present illness is due to the fact that a few days ago I was caught in a shower and got my feet wet."

Upon examination I found slight dullness over the left axillary region with only an appreciable diminution of the respiratory sounds of the same side. These normal modification sounds were so very slight, that it was with much difficulty I determined upon their presence. My diagnosis was pleurisy, and her treatment was sudorific, and directed to the prevention of serous exhalation.

On the following day her married daughter reported her mother was much improved and very comfortable. For five or six days she continued to convalesce, and very promising hopes were entertained of her speedy and complete recovery.

Upon the seventh day her condition was much worse, quite like that at the time of my first visit; a similar treatment was pursued with similar results for at least a few days.

Subsequent to this date the patient did not come under my observation till May 18th, when I learned that for the first time she found it necessary to remain in bed.

Inspection of her chest showed marked deformity, the right side was bulging and prominent, with a corresponding depression of the left. This was so great that the spinal column participated in the deformity, there being marked lateral curvature. The respiratory sounds of the right lung were exaggerated but not otherwise abnormal, while those of the left could scarcely be heard. There was complete flatness below the third rib. A hypodermic needle was thrust into the pleural cavity and immediately filled with a clear straw colored serum. Upon the same day thorocentesis was performed, and by estimate fully three pints of serum were removed. The withdrawal of the instrument was accompanied with coughing so common under such circumstances.

There was nothing unnatural in the appearance of the serum as differing from ordinary pleuritic effusion.

Much relief was experienced after its removal, the patient breathing much easier and expressing herself as feeling very comfortable. The side was secured by adhesive plaster in the usual manner. Three or four days later it was evident that she was gradually losing strength and becoming more feeble. Emaciation progressed very rapidly. She coughed occasionally and expectorated brownish sputa in small quantity.

Seven days after the first operation I again operated, removing this time about one pint. The fluid was a little muddy and mixed with blood. On removing the trocar there was considerable hemorrhage. I covered the point of puncture with plaster and applied collodion.

This did not seem to control the hemorrhage only for a few seconds, when it gushed from beneath the plaster and ran down her side in a stream. I began to feel guilty of having punctured the intercostal artery. I then passed a strip of adhesive plaster nearly around her body making a considerable pressure upon the seat of hemorrhage which effectually controlled it. I waited expecting the vessel might empty its contents into the pleural cavity, but this did not occur. After the withdrawal of this fluid the patient seemed to feel somewhat relieved, but her comfort was only temporary, lasting but a few hours. From this date her dyspnœa rapidly increased and she became more and more exhausted till the time of her death, June 7th, ten days after the second operation.

From the date of the first operation she complained of pain in her left shoulder and at the point of puncture, but during her whole illness the pain was not excrutiating, one grain of morphine in divided doses being sufficient to control it for a week. For two or three weeks before the disease terminated fatally her pulse was feeble and rapid, beating from 120 to 130 per minute. Her emaciation was rapidly progressive, and the skin presented a yellow tint. She had night sweats, occasional diarrhea and febrile paroxysms with chills and palpitation, and as before stated, some cough without much expectoration.

These symptoms, with her family history, led me to suspect the presence of phthisis, but I was unable to satisfy myself that such was the case. From the date of the second operation to the time of her death the nature of the case remained in doubt. To reach a more satisfactory explanation, a post-mortem was solicited and secured. Dr. Pettit and myself carefully removed the lungs. The left was firmly adherent to the thoracic walls both posteriorly and laterally, and was removed with much difficulty. A portion of the ribs and a part of the pericardium were also removed and left in their normal relations to each other. The left lung, except at the extreme apex and internal lateral border is a solid cancerous mass—scarcely a vestage of it capable of receiving air during the act of respiration, the bronchial tubes participating in the diseased action. The lung was submerged in the pleuritic fluid, and is a remarkable instance of an organ being infiltrated with a new formation, and at the same time diminished in size. The right lung was greatly dilated and filled with nodular deposits in sizes varying from a pea to a walnut, but none of them broken down. The cardiac surface of the pericardium is smooth, and on inspection presents no nodular growth. Upon pressure however, distinct small nodules can be felt, they are external to the walls of the sack although attached to it. The heart we thought to be rather small but otherwise healthy. Louis observed that the hearts of persons dying of cancer of the lungs, were below the average size.

Although it was our intention, we regret not having examined the condition of other organs, but judging from the patient's history, there being no evidence of malignant disease existing elsewhere with the pathological anatomy it seems reasonable and fair to presume the left lung or pleural surface to have been the seat of the disease.

Physical examination has contributed its share of information when it tells us whether the lung be solidified wholly or in part, and whether the pleural cavity contains fluid. Beyond that it offers no definite information. A lung may contain a large number of cancerous nodules without producing suspicious sounds, except to exaggerate the normal ones. The absence of pain is quite characteristic of cancer in this situation, and it is possible that the act of puncturing the diseased mass caused some of the pain she experienced. The hemorrhage following the second effort at paracentesis came from the cancerous mass into which the trocar was carried. The only symptoms that would be likely to lead us to suspect malignant disease in the case reported, were the advanced age of the patient, the peculiar tint of the skin or cancerous habit, and rapid emaciation, but these associated with less important ones would not in any single case be sufficient to fix a diagnosis.

Rokitansky once advanced the opinion that tuberculosis and cancer did never coexist, but later, modified the statement and said that they rarely coexist. This woman is believed to be an offspring of tuberculous parents, has borne tubercular children.

Cancer of the lungs is rarely observed, and then it usually appears as secondary deposit and is comparatively easy of diagnosis. But in cases when cancerous disease is not found to exist in other parts of the body, and the symptoms direct attention to the pulmonary organs as in the case reported, it becomes difficult of diagnosis, and the majority of cases Reynolds says, admit of no diagnosis.

*Medullary Carcinoma* or the *Encephaloid* form is the variety usually attacking the pulmonary organs. *Scirrhus* is said to be rare, and *Colloid* almost never seen.

